# Hologram QSAR Models of 4-[(Diethylamino)methyl]-phenol Inhibitors of Acetyl/Butyrylcholinesterase Enzymes as Potential Anti-Alzheimer Agents

**DOI:** 10.3390/molecules17089529

**Published:** 2012-08-09

**Authors:** Simone Decembrino de Souza, Alessandra Mendonça Teles de Souza, Ana Carolina Corrêa de Sousa, Ana Carolina Rennó Sodero, Lúcio Mendes Cabral, Magaly Girão Albuquerque, Helena Carla Castro, Carlos Rangel Rodrigues

**Affiliations:** 1 Laboratory of Molecular Modeling & QSAR-3D (ModMolQSAR), Faculty of Pharmacy, Federal University of Rio de Janeiro (UFRJ), Rio de Janeiro, 21949-900, RJ, Brazil; 2 Laboratory of Industrial Pharmaceutical Technology (LabTIF), Faculty of Pharmacy, Federal University of Rio de Janeiro (UFRJ), Rio de Janeiro, 21941-590, RJ, Brazil; 3 Laboratory of Molecular Modeling (LabMMol), Program of Post-Graduation in Chemistry (PPGQu) Institute of Chemistry, Federal University of Rio de Janeiro (UFRJ), Rio de Janeiro, 21949-900, RJ, Brazil; 4 Laboratory of Antibiotics, Biochemistry, Education and Molecular Modeling (LABiEMol), Institute of Biology (IB), Fluminense Federal University (UFF), Campus of Valonguinho, Niterói, 24210-130, RJ, Brazil

**Keywords:** HQSAR, acetylcholinesterase, butyrylcholinesterase, Alzheimer’s disease (AD)

## Abstract

Hologram QSAR models were developed for a series of 36 inhibitors (29 training set and seven test set compounds) of acetyl/butyrylcholinesterase (AChE/BChE) enzymes, an attractive molecular target for Alzheimer’s disease (AD) treatment. The HQSAR models (N = 29) exhibited significant cross-validated (AChE, q^2^ = 0.787; BChE, q^2^ = 0. 904) and non-cross-validated (AChE, r^2^ = 0.965; BChE, r^2^ = 0.952) correlation coefficients. The models were used to predict the inhibitory potencies of the test set compounds, and agreement between the experimental and predicted values was verified, exhibiting a powerful predictive capability. Contribution maps show that structural fragments containing aromatic moieties and long side chains increase potency. Both the HQSAR models and the contribution maps should be useful for the further design of novel, structurally related cholinesterase inhibitors.

## 1. Introduction

Alzheimer’s disease (AD) is the most common cause of dementia in elderly people. As the worldwide population ages, AD is reaching epidemic levels, giving rise to a huge human, social, and economic burden [1,2]. AD is neuropathologically characterized by the extracellular deposition of amyloid plaques, as well as intracellular neurofibrillary tangles (NFTs) [3,4]. However, the cause of AD is still a subject of considerable discussion, and there are no available treatments that stop or reverse disease progression. Current treatments only help alleviate AD symptoms [5].

The main therapeutic approaches in AD treatment are based on the cholinergic hypothesis. According to this hypothesis, the neurodegenerative processes lead to a selective destruction of the cholinergic neurons, resulting in a lack of central cholinergic transmission. This deficiency causes cognitive declination, a loss of ability to perform daily tasks, reduced attention, and a loss of memory [6,7]. Thus, the symptomatic treatment of AD is based on acetylcholinesterase (AChE) inhibitors, which have positive effects on the cognitive, functional, and behavior symptoms of the disease and consequently increase acetylcholine levels and cholinergic neurotransmission in the brain [8]. The increase in cholinergic transmission by the use of drugs that enhance the central cholinergic function is currently thought to be the best approach for the treatment of AD [9].

Butyrylcholinesterase (BChE), another enzyme that hydrolyzes acetylcholine, is predominantly found in the glia neurons and exists in greater amounts than the progressively decreasing AChE in the brains of AD patients. Studies with BChE may hold promise in the search of new inhibitors for AD treatment [10].

Further studies using molecular modeling predictive techniques, such as hologram quantitative structure-activity relationship (HQSAR) [11], in conjunction with experimental techniques, such as X-ray 3D structure elucidation, may help uncover new human cholinesterase inhibitors as potential anti-Alzheimer agents. HQSAR, as with other fragment-based methods, uses fragment fingerprints [12] as a predictive variable of the biological activity variation or other related data. It is a method that develops statistically validated QSAR models [13] using topological (2D) parameters such as hologram length and fragment distinctions [14,15].

In an attempt to design new chemical entities with efficient anticholinesterase activities, we have explored the 2D molecular features of a series of 4-(diethylamino)methyl]-phenol AChE/BChE inhibitors [16] using the HQSAR method, a powerful ligand-based strategy in drug design [17].

## 2. Results and Discussion

### 2.1. HQSAR Analysis

A data set of 36 4-[(diethylamino)methyl]-phenol AChE/BChE inhibitors was compiled from the work of Yu *et al.* [16]. The chemical structures and biological data of these AChE/BChE inhibitors are listed in Table 1, as well as the distribution in the training set (29 compounds) and test set (seven compounds), an important step in the development of QSAR models aiming to maximize the test set diversity and to analyze the model prediction accuracy [18].

**Table 1:** Chemical structures and biological data (pIC_50_, M) of the AChE and BChE inhibitors. 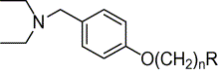

#	n	R	pIC_50_^a^			#	n	R	pIC_50_ [16]	
			AChE	BChE					AChE	BChE
1	2	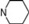	4.86	4.99		19	6	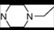	5.79	5.66
2	2	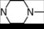	4.94	4.69		20	6	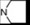	6.02	5.77
3	4	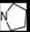	5.47	5.85		21	6	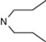	5.67	5.87
4	4	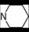	5.17	6.14		22*	**6**	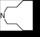	**6.17**	**6.64**
5	4	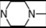	5.25	5.01		23	8	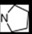	7.08	7.82
6 *	**4**	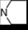	**4.97**	**5.56**		24	8	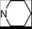	7.04	8.13
7	4	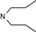	4.86	4.93		25	8	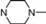	6.68	7.96
8	4	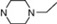	4.99	5.60		26	8	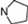	7.11	7.85
9	5	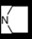	6.30	5.81		27*	**8**	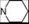	**6.85**	**7.31**
10	5	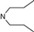	5.37	6.17		28	8	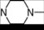	6.77	8.04
11 *	**5**	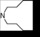	**5.61**	**5.03**		29	8	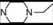	6.85	7.77
12	5	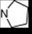	5.75	5.51		30 *	**10**	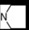	**6.52**	**7.17**
13	5	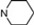	5.08	5.35		31	10	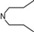	6.22	7.11
14	5	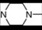	5.25	5.75		32	10	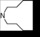	6.27	7.48
15	5	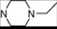	5.61	5.72		33*	**10**	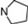	**5.24**	**6.72**
16	6	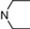	6.36	5.89		34	10	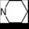	6.51	7.57
17	6	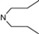	6.05	5.86		35	10	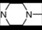	6,47	7.58
18	6	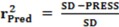	5.74	5.42		36	10	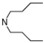	6.59	7.82

* Test set compounds.

HQSAR investigations require selecting values for the parameters that specify the hologram length, as well as the size and type of fragments to be encoded. The generation of the molecular fragments was performed using the following fragment distinction parameters: atoms (A), bonds (B), connections (C), hydrogen (H) atoms, chirality (Ch), and donor/acceptor (DA) atoms. For both the AChE and BChE inhibitor training sets, a default fragment size (4–7) was employed to obtain relevant statistical indexes with different combinations of fragment distinction parameters (Table 2). To improve these statistical indexes (Table 2), we investigated the influence of fragment size (2–5, 3–6, 4–7, 5–8, 6–9, 7–10, 8–11, and 9–12) (Table 3) on the fragment distinction parameters of those models having the highest statistical indexes.

**Table 2:** Summary of HQSAR statistical indexes for various fragment distinction (FD) parameters using the fragment size default (4–7) for both AChE and BChE inhibitors.

	AChE Statistical Indexes ^a^							BChE Statistical Indexes ^a^					
FD ^b^	q^2^	r^2^	SE	SEcv	NC	HL		q^2^	r^2^	SE	SEcv	NC	HL
**A/B**	0.660	0.833	0.328	0.468	6	59		**0.706**	**0.780**	**0.545**	**0.630**	**2**	**61**
**A/C**	0.642	0.833	0.328	0.480	6	307		0.696	0.777	0.549	0.640	2	199
**A/H**	0.635	0.785	0.364	0.475	5	53		0.704	0.754	0.566	0.621	1	97
**A/DA**	0.655	0.826	0.321	0.452	4	71		0.656	0.761	0.568	0.682	2	401
**B/C**	**0.674**	**0.787**	**0.348**	**0.431**	**3**	**307**		0.636	0.753	0.588	0.715	3	307
**B/H**	0.657	0.732	0.383	0.434	2	59		0.656	0.733	0.600	0.682	2	307
**C/H**	0.672	0.783	0.358	0.456	3	307		0.637	0.750	0.592	0.713	3	53
**C/DA**	0.638	0.811	0.335	0.463	4	97		0.676	0.792	0.530	0.661	2	59
**A/B/C**	0.643	0.830	0.324	0.469	5	401		0.690	0.777	0.548	0.647	2	199
**A/C/H**	0.624	0.782	0.367	0.482	5	257		0.694	0.744	0.578	0.631	1	61
**A/B/C/H**	**0.619**	**0.781**	**0.368**	**0.485**	**5**	**257**		**0.693**	**0.743**	**0.578**	**0.632**	**1**	**61**

**^a^** q^2^, LOO cross-validated correlation coefficient; r^2^, non cross-validated correlation coefficient; SEcv, cross-validated standard error; SE, non cross-validated standard error; NC, optimal number of components; HL, hologram length; **^b^** Fragment distinction parameters: atoms (**A**), bonds (**B**), connections (**C**), hydrogen (**H**) atoms, and donor/acceptor (**DA**) atoms.

**Table 3:** Summary of HQSAR statistical indexes for the influence of various fragment sizes (FS) using the fragment distinction parameters B/C and A/B for AChE and BChE inhibitors, respectively.

	AChE Statistical Indexes ^a^							BChE Statistical Indexes ^a^					
FS	q^2^	r^2^	SE	SE_cv_	NC	HL		q^2^	r^2^	SE	SE_cv_	NC	HL
**2–5**	0.598	0.764	0.390	0.509	6	353		0.703	0.768	0.560	0.633	2	53
**3–6**	0.605	0.758	0.379	0.484	4	307		0.705	0.775	0.557	0.631	2	53
**4–7**	0.674	0.787	0.348	0.431	3	307		0.706	0.780	0.545	0.630	2	61
**5–8**	0.695	0.810	0.329	0.417	3	61		0.862	0.957	0.272	0.489	8	401
**6–9**	0.698	0.815	0.324	0.415	3	97		0.880	0.959	0.268	0.456	8	61
**7–10**	0.693	0.813	0.326	0.418	3	59		0.894	0.966	0.244	0.430	8	199
**8–11**	0.782	0.940	0.202	0.383	7	307		0.893	0.961	0.250	0.412	6	151
**9–12**	**0.787**	**0.965**	**0.156**	**0.388**	**8**	**151**		**0.904**	**0.952**	**0.265**	**0.373**	**4**	**151**

^a^ q^2^, LOO cross-validated correlation coefficient; r^2^, non-cross-validated correlation coefficient; SEcv, cross-validated standard error; SE, non-cross-validated standard error; NC, optimal number of components; HL, hologram length.

Finally, the best HQSAR model for the AChE inhibitors was generated using bonds (B) and connections (C) as fragment distinction parameters and 9–12 as the fragment size, showing q^2^ = 0.787 and r^2^ = 0.965 (Table 3). For the BChE inhibitors, the best HQSAR model was developed using atoms (A) and bonds (B) as fragment distinction parameters and 9–12 as the fragment size, showing q^2^ = 0.904 and r^2^ = 0.952 (Table 3).

It is interesting to note that the best model of each data set (AChE and BChE inhibitors) was generated when long fragments (*i.e.*, 9–12 for AChE and BChE) were considered. Notably, there is a direct relationship between the side chain length and the inhibitory potency where a length of eight methylene units seems to be optimal [1]. Therefore, the generation of models with a long fragment size was not unexpected.

As an additional validation test, we performed the randomization of the biological activity values (*i.e.*, Y-randomization test) and re-run the HQSAR analysis for the AChE and BChE training sets, resulting in q^2^ values ranging from 0.083 to 0.191 and −0.113 to −0.133, respectively. This result unsure the robustness of the original HQSAR regression models, since there are no chance correlations.

In HQSAR, the structure encoded within a 2D fingerprint can be directly related to biological activity variation, and the most important test of a QSAR model is its ability to predict the activity value for new compounds. The external validation of the HQSAR models was performed by predicting the biological potencies of the test set using the best models derived from the training set. The predictive ability of the HQSAR models is expressed with predictive r^2^ values, which is similar to cross-validated r^2^ (q^2^), and is defined using the Equation (1):


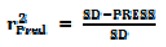
(1)


In Equation (1), **SD** is the sum of squared deviations between the biological activity of the test set and the mean activity of the training set molecules, and **PRESS** is the sum of squared deviations between the observed and the predicted activity values for every molecule in the test set [19]. 

**Figure 1:**
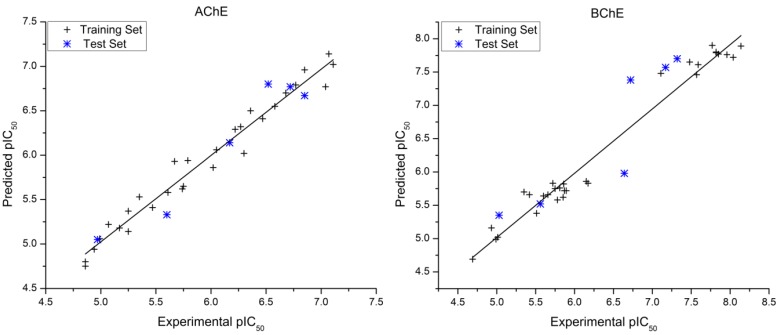
Plot of experimental *versus* predicted pIC_50_ values of the training and test sets of the AChE and BChE inhibitors.

The experimental pIC_50_, predicted pIC_50_ and residual (pIC_50_Exp. − pIC_50_Pred.) values of the AChE and BChE inhibitors obtained by the best HQSAR model for each data set are reported in Table 4. The comparison plots between the experimental and predicted potencies of both the training and test sets for the AChE and BChE inhibitors, using the best HQSAR models, are shown in Figure 1.

In this work, we considered as outliers all compounds of the training and test sets where the residual value exceeded twice the standard error of estimate of the model [20] (AChE, 2*SD = 0.30; BChE, 2*SD = 0.52). According to this criterion, there are two outlier compounds for the BChE model: compound **33**, which presented the highest residual values in BChE (residual = −0.66), and compound **22**, BChE (residual = 0.66), but none of them presented residual values higher than one logarithmic unit. Compounds **33** and **22** belong to the test set and did not take part in the model building process. The outlier **33** is analogous to **26** (the most potent AChE inhibitor and the fourth most potent BChE inhibitor), and both have the same ethylpiperazine R-group moiety, differing only in their side chain lengths (Table 1). The HQSAR models overestimated the biological potency of **33** because of the direct relationship between the number of methylene spacer units in the side chain and the inhibitory potency of this series, as discussed earlier [1]. However, for this particular series, there is an optimum side chain size of eight methylene units, instead of 10 units as in compound **33**. The outlier **22** is analogous to **21**, and both have the same chain length but differ in the number of methylene units in their R-group moieties. The HQSAR models underestimated the biological potency of **22** because this derivative has two more methylene units than compound **21**, resulting in a potency estimate closer in value to **21**.

**Table 4:** Experimental and predicted pIC_50_ (M) values and residuals (pIC_50_Exp. − pIC_50_Pred.) of AChE and BChE inhibitors using the best HQSAR models.

#		AChE pIC_50_		Residual				BChE pIC_50_		Residual
		Exp. ^a^	Pred.					Exp. [16]	Pred.	
**1** *		4.86	4.8	0.06				4.99	4.99	0.00
**2**		4.94	4.94	0.00				4.69	4.69	0.00
**3**		5.47	5.41	0.06				5.85	5.62	0.23
**4**		5.17	5.18	−0.01				6.15	5.86	0.29
**5**		5.25	5.37	−0.12				5.01	5.02	−0.01
**6** *		4.97	5.05	−0.08				5.56	5.52	0.04
**7**		4.86	4.75	0.11				4.93	5.16	−0.23
**8**		4.99	5.06	−0.07				5.60	5.64	−0.04
**9**		6.3	6.02	0.28				5.81	5.76	0.05
**10**		5.35	5.53	−0.18				6.17	5.83	0.34
**11** *		5.60	5.33	0.27				5.03	5.35	−0.32
**12**		5.75	5.65	0.10				5.51	5.38	0.13
**13**		5.07	5.22	−0.15				5.35	5.70	−0.35
**14**		5.25	5.14	0.11				5.75	5.75	0.00
**15**		5.61	5.58	0.03				5.72	5.83	−0.11
**16**		6.36	6.5	−0.14				5.89	5.72	0.17
**17**		6.05	6.06	−0.01				5.86	5.82	0.04
**18**		5.74	5.62	0.12				5.42	5.66	−0.24
**19**		5.79	5.94	−0.15				5.66	5.66	0.00
**20**		6.02	5.86	0.16				5.78	5.58	0.20
**21**		5.67	5.93	−0.26				5.87	5.72	0.15
**22** *		6.17	6.14	0.03				6.64	5.98	0.66
**23**		7.07	7.14	−0.07				7.82	7.80	0.02
**24**		7.04	6,77	0.27				8.14	7.89	0.25
**25**		6.68	6.7	−0.02				7.96	7.76	0.20
**26**		7.11	7.02	0.09				7.85	7.77	0.08
**27** *		6.85	6.67	0.18				7.32	7.70	−0.38
**28**		6.77	6.79	−0.02				8.04	7.72	0.32
**29**		6.85	6.96	−0.11				7.77	7.90	−0.13
**30** *		6.52	6.80	−0.28				7.17	7.57	−0.40
**31**		6.22	6.29	−0.07				7.11	7.48	−0.37
**32**		6.27	6.32	−0.05				7.48	7.65	−0.17
**33** *		6.72	6.77	−0.05				6.72	7.38	−0.66
**34**		6.47	6.41	0.06				7.57	7.46	0.11
**35**		6.58	6.55	0.03				7.59	7.61	−0.02
**36**		4.86	4.8	0.06				7.82	7.79	0.03

* Test set compounds.

**Figure 2:**
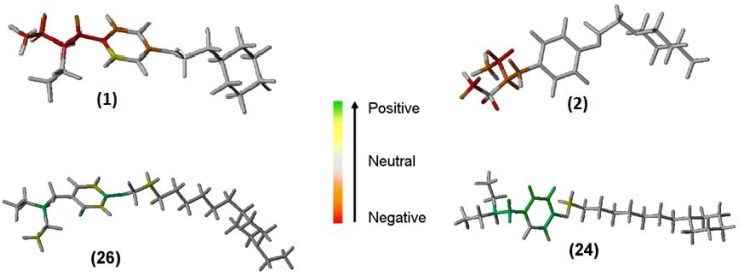
The HQSAR contribution maps of compounds **26** (most potent) and **1** (least potent) for the AChE inhibitory activity, and **24** (most potent) and **2** (least potent) for BChE inhibitory activity.

A complete HQSAR analysis involves the investigation of important molecular fragments directly related to the biological activity variation so that one may propose structural modifications. Thus, the HQSAR models can be graphically displayed as color-coded structure diagrams in which the color of each atom reflects its contribution to the potency variation. The red and green ends of the spectrum reflect negative and positive contributions, respectively whereas atoms with intermediate contributions are colored white [15]. The individual atomic contributions of the most (compounds **26** and **24**) and least (compounds **1** and **2**) potent AChE and BChE inhibitors, according to the best HQSAR models, are displayed in Figure 2.

The HQSAR contribution maps for AChE and BChE inhibitors show that the structural fragment containing aromatic moieties increases potency, reinforcing the importance of the aromatic system in establishing π-π stacking interactions with the aromatic residues present in both enzymes, as described elsewhere [16,21,22]. In addition, the HQSAR map obtained from the BChE inhibitors also revealed the importance of protonation of the amine nitrogen atom. This chemical group is important, as it is involved in electrostatic interactions, also in agreement with literature data [16].

The compounds of this series have been designed to behave as AChE/BChE dual site inhibitors, *i.e.*, binding simultaneously to the catalytic anionic site (CAS) and the peripheral anionic site (PAS) [16]. For AChE inhibitors, the side chain length was another feature highlighted in the HQSAR map contribution, emphasizing the need for a long side chain fragment. In fact, the crystal structure of AChE from *Torpedocalifornia* shows that the CAS is located at the bottom of a deep and narrow gorge composed of 14 aromatic residues, and the PAS is located at the entrance of this gorge at a distance of ~20 Å [16,23]. Moreover, the estimated CAS-PAS distance is ~14 Å [24].

Surprisingly, a comparison of the contribution maps of the most (**26**) and least (**1**) potent AChE inhibitors revealed that the positive and negative contributions to biological activity come from the fragment that is common to both molecules (Figure 2). The same can be seen in the most (**24**) and least (**2**) potent BChE inhibitors. A possible explanation for this finding is that the compounds with a longer spacer group can reach both CAS and PAS simultaneously, enhancing binding. Conversely, compounds with shorter linkers cannot reach both binding sites and, thus, can bind only weakly, being readily displaced by water molecules. 

## 3. Experimental

### 3.1. Data Set and Molecular Modeling

The data set used for the HQSAR studies contains the 36 4-[(diethylamino)methyl]-phenol derivatives developed by Yu *et al.*, showing cholinesterase inhibitory activity against both AChE and BChE enzymes [16]. The biological activity of all compounds, originally expressed as IC_50_ (µM) values [16], were converted to pIC_50_ (M) (−Log IC_50_, Table 4) values (Table 1). The chemical structures of all compounds were constructed using the PC Spartan’10 program [25].

HQSAR

HQSAR modeling was performed using the SYBYL 8.0 package [26]. The 36 compounds were divided in the same training (29 compounds) and test (seven compounds) sets for both the AChE and BChE studies, considering that test set molecules should represent high, middle and low potency compounds, also spanning structural diversity, to avoid potential problems during the HQSAR model external validation.

The HQSAR study has three key steps: the generation of fragments for each molecule in the training set, the encoding of these fragments in holograms, and the correlation with available biological data [27]. Parameters that are associated with the generation of holograms, such as hologram length (HL), fragment size, and fragment distinction, may affect the HQSAR model; thus, different combinations of these parameters were considered during the HQSAR runs [14,15].

The structures of the phenolic derivatives, which comprise the training set, were converted to structural fragments initially using a minimum and maximum number of four and seven atom fragments, respectively. All fragments produced were allocated to the defined size of the molecular hologram (53, 59, 61, 71, 83, 97, 151, 199, 257, 307, 353, 401 bins), and analysis was performed to distinguish the fragments based on a combination of two or more of the following parameters: atoms (A), bonds (B), connectivity(C), hydrogen (H) atoms, chirality (Ch), and donor/acceptor (DA) atoms. The influence of the fragment size on the statistical indexes was evaluated using the best combinations of distinguishing fragments.

All QSAR models were generated using partial least squares (PLS), and the internal validation was performed by leave-one-out (LOO) cross-validation (q^2^). In order to access the risk of chance correlation, it has been carried out the y-randomization (y-scrambling or response randomization) test, an additional validation procedure, by randomization the biological activity values (*i.e.*, Y-randomization or Y-scrambling test) and re-run the HQSAR analysis for the AChE and BChE training sets. An external validation was performed with the test set compounds, which was not considered for the HQSAR model development. The predictive ability of the models was investigated by predictive r^2^ values (r^2^pred). The parameters used above were applied to both AChE and BChE studies.

## 4. Conclusions

HQSAR models were obtained using the same training (29 compounds) and test (seven compounds) sets for all analyses and demonstrated reliable predictive power for both AChE and BChE inhibitors. These models (N = 29) showed significant cross-validated (AChE, q^2^ = 0.787; BChE, q^2^ = 0.904) and non-cross-validated (AChE, r^2^ = 0. 965; BChE, r^2^ = 0.952) correlation coefficients. The strong correlation between the experimental and predicted potency values for the test set compounds (external validation) shows the reliability of the constructed HQSAR models for both studies. HQSAR contribution maps of these models were used to explain the importance of the structural fragment to the overall activity of this series; structural fragments containing aromatic moieties and long side chains increase potency, in agreement with previous works. Therefore, the HQSAR models and contribution maps should be useful for future efforts in the design of new cholinesterase inhibitors with improved biological activity.
